# Bingeing on fat increases cocaine reward

**DOI:** 10.18632/oncotarget.15260

**Published:** 2017-02-10

**Authors:** M. Carmen Blanco-Gandía, Marta Rodríguez-Arias

**Affiliations:** Departamento de Psicobiología, Unidad de Investigación Psicobiología de las Drogodependencias, Facultad de Psicología, Universitat de València, Valencia, Spain

**Keywords:** cocaine, fat, binge eating, gene expression, reward, Neuroscience

In recent years, rates of overeating have been increasing dramatically, especially among the younger population. Consumption of fast and processed food plays an important role in cardiovascular diseases, diabetes and obesity. Binge eating is one of the most common eating disorders. According to the fifth edition of the Diagnostic and Statistical Manual of Mental Disorders, binge eating is a specific form of overeating, characterized by intermittent excessive eating in a short period of time and marked by feelings of lack of control. Characteristically, binge eating is not driven by metabolic needs and occurs in the absence of food restriction. Foods that are consumed during a binge episode are typically high in calories, fat and/or sugar. Although it is related to obesity, many people who binge eat are not obese, and most obese people do not present binge eating disorders [1]. Many teenagers display this pattern of hedonic eating without fulfilling the clinical criteria for binge-eating disorder. During adolescence, a critical period of brain maturation, individuals are especially vulnerable to harmful influences such as inadequate dietary habits or drug abuse.

The alteration of dopamine brain levels, a neurotransmitter critical to the reward process, is a common neurobiological mechanism implicated in obesity and drug addiction. As with drugs of abuse, the ingestion of palatable foods rich in sugar or fat, activates dopaminergic neurons within reward centers. The escalation of palatable food consumption observed in binge eating mimics what occurs in addiction, since there is a transition from controlled to compulsive intake and subsequent loss of control. In fact, subjects with substance abuse disorders and obese individuals show reduced levels of dopaminergic receptors in the nucleus accumbens, a critical structure of the reward system, as a compensatory response to the excessive dopaminergic neurotransmission [2].

Drug use during adolescence often predicts an increased likelihood of continued use into adulthood [3] and due to the common neurobiological pathways that stimulate fat intake and drugs of abuse, several studies have suggested that high consumption of palatable food could also increase vulnerability to drug use. Epidemiological studies have reported that, in clinical populations there is an overlap between binge-eating disorders and drug addiction [4], as both are characterized by typical addictive processes such as tolerance, withdrawal and compulsive food/drug-seeking. Several studies performed in rodents exposed to high-sugar diets have reported increased effects of cocaine and ethanol [5]. Although less studied, we know that a continuous high-fat diet seems to decrease cocaine reward [6], but restricted access to this diet increases the locomotor effect of cocaine in adolescent mice [7].

In order to better understand the effects of intermittent access to fat during adolescence, we have examined the rewarding effects of cocaine in mice that binged on fat during adolescence [8]. Adolescent mice had limited access to a specific high-fat chow for 2 h, three times a week (on Monday, Wednesday and Friday), while they had constant access to standard chow. We observed an increase in the amount of fat consumed by these animals within this period, and an escalation of intake within the second week of fat exposure. Therefore, the limited access protocol led to the development of fat-bingeing behaviors. However, there was no increase in body weight or leptin levels in the group exposed to fat, although ghrelin was decreased. Leptin and ghrelin are two hormones that influence energy balance. Leptin suppresses food intake and induces weight loss. Ghrelin, on the other hand, is known as the hunger hormone, as it is secreted when the stomach is empty.

To evaluate the rewarding effects of cocaine, we employed the conditioned place preference (CPP), which assesses the role of the environmental cues associated with the drug, and the intravenous self-administration (SA) procedure, which evaluates the hedonic properties of drugs of abuse. Mice that binged on fat during adolescence developed CPP with a low dose that was not effective in regular fed animals, presented a stronger memory of the environmental cues associated with cocaine, and also relapsed in seeking the drug even after the loss of the drug-associated memory. Comparable results were obtained in the SA procedure. The total amount of cocaine consumed increased in mice exposed to the high-fat diet, and, after fat withdrawal, exposure to a new fat binge reinstated cocaine seeking. Therefore, intermittent fat ingestion increases sensitivity to the rewarding effects of cocaine and also heightens vulnerability to relapse in seeking this drug.

**Figure 1:**
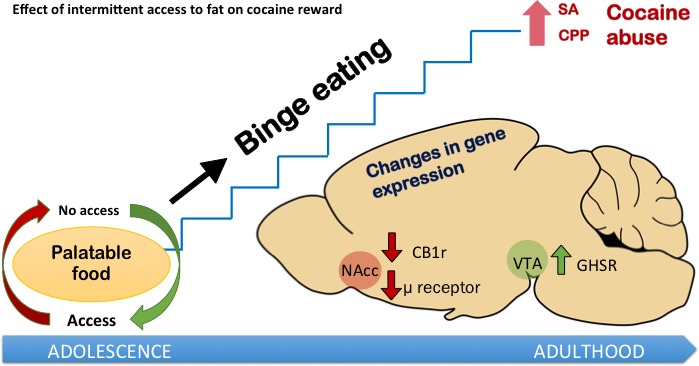
Intermittent fat ingestion increases sensitivity to the rewarding effects of cocaine and modifies the opioid and endocannabinoid systems

Intermittent fat ingestion, although it does not induce relevant hormonal changes, alters key neurotransmitter systems involved in drug addiction and food ingestion in relevant brain structures that control both processes (Figure 1). Gene expression of cannabinoid CB1 and mu opioid receptors were decreased in the nucleus accumbens of fat fed mice, accompanied by an increased expression of ghrelin receptor in the ventral tegmental area. Thus, high-fat bingeing modulates not only the dopaminergic system, but also the opioid and endocannabinoid systems. This makes animals more prone to seeking and consuming cocaine. Our results suggest that the fat composition of a diet and the way in which fat is consumed plays an important role in determining the sensitivity of an individual drug abuse, particularly during adolescence.
